# Hardiness personality and mental health of financially-struggling medical students in private universities in China: the intervening roles of coping styles and gender

**DOI:** 10.3389/fpubh.2024.1458049

**Published:** 2024-11-04

**Authors:** Huan Liu, Jiabao Chen, Qinghe Peng, Hao Zhang, Yating He

**Affiliations:** ^1^College of Pharmaceutical Economics and Management, Anhui University of Chinese Medicine, Hefei, China; ^2^Key Laboratory of Philosophy and Social Science of Anhui Province on Data Science and Traditional, Chinese Medicine Innovation and Development, Anhui University of Chinese Medicine, Hefei, China; ^3^College of Humanities and International Exchange, Anhui University of Chinese Medicine, Hefei, China; ^4^College of Integrated Traditional Chinese and Western Medicine, Anhui University of Chinese Medicine, Hefei, China; ^5^Guang’anmen Hospital, China Academy of Chinese Medical Sciences, Beijing, China

**Keywords:** mental health, coping style, hardiness personality, private universities, financially-struggling medical students

## Abstract

**Background:**

Chinese adolescents are at higher risk of depression, especially the mental health problems of financially disadvantaged medical students, which are significantly higher than those in other age groups, which brings great challenges to the mental health workers in universities.

**Methods:**

1,280 medical students with a family poverty background in China completed a questionnaire on hardiness personality, coping style and mental health. After descriptive statistics and Pearson correlation analysis between hardiness personality, coping style and mental health levels, we tested the mediation of coping style and the moderating effects of gender.

**Results:**

Hardiness personality significantly positively affected the financially-struggling medical student’s mental health level. Positive coping style had a significant positive impact on hardiness personality and mental health level, while negative coping style had a significant negative effect on mental health level. Positive coping and negative coping are the mediators between financially-struggling medical students’ hardy personalities and mental health levels. In medical students with a family poverty background, gender plays a regulatory role in coping style and mental health levels. In medical students with a family poverty background, gender plays a moderate role in coping style and mental health levels.

**Conclusion:**

This study adds some knowledge about the effects of hardiness personality on individual mental health. It makes new recommendations for improving the mental health status of vulnerable groups, while it can support future investigations by scholars and educators on how to improve the mental health of students under learning and financial-related stress.

## 1 Introduction

Globally, the prevalence of mental health problems has risen recently. In particular, the mental health issues that post-epidemic college students are facing provide a new challenge to the field of public health (1). According to research, medical students are a special social group that is vulnerable to mental illnesses, psychological stress, and a decline in life satisfaction in many nations (2–5). Medical students’ anxiety and depression are exacerbated by financial difficulties, sleep deprivation, job stress, and academic pressure (6–9). More financial and social constraints are placed on financially-struggling medical students in China than on other medical students (10). Research by Azim SR et al. was carried out in Pakistani private medical university. Researchers discovered that students from medium and lower socioeconomic groups felt more psychologically distressed than students from higher socioeconomic classes. According to Wege N et al., there is a substantial correlation between mental health illnesses and symptoms and projected economic issues (11). Furthermore, compared to their classmates, Medical students with a family poverty background face a more significant social status gap, which erodes their sense of community and social support system and has a detrimental effect on their development and interpersonal skills (12). The assertion is consistent with Cheng Lan’s study (13). Meanwhile, private university students typically display lesser comprehensive quality, a weakened sense of identity, and more psychological strain than students attending public institutions. The demanding study requirements and stringent evaluation standards of medical school have been the subject of several research studies, which have demonstrated that they are a major cause of psychological stress in and of themselves (14, 15). Financially-struggling medical students may struggle with feelings of self-worth and expected conflicts while they pursue academic and professional achievement, which exacerbates psychological issues for these students. Medical students may experience negative outcomes from prolonged psychological stress, such as subpar academic performance, dropping out of school, abusing alcohol or other drugs, and having suicidal thoughts and attempts (16–20). In this context, individual personality traits and coping styles are crucial factors affecting mental health. A hardy personality helps individuals cope effectively with life and promotes mental health and well-being. The type of coping that individuals choose influences their overall quality of life. Therefore, on the one hand, this study constructs a more comprehensive model to describe the interrelationship between hardiness personality, coping style and mental health, promotes the development of relevant theories in the field of psychology, and on the other hand, reveals the positive impact of hardiness personality on mental health, which can provide new perspectives for psychological intervention and treatment, to improve the ability of individuals to cope with stress and challenges.

### 1.1 Hardiness personality and mental health

Kobasa (21) used the term “hardiness personality” to describe a group of attitudes, convictions, and behavioral patterns that shield people from disease as they deal with stressors in life and at work (22). According to Soderstrom Studies, persons with high hardiness typically utilize constructive coping mechanisms and exhibit fewer symptoms of both mental and physical illnesses. In contrast, those with low tenacity normally resort to avoidance techniques and are more vulnerable to both (23). Academic studies on the connection between mental health and toughness personality are divided. Stefanie and Graham have shown hardiness to regulate stress and wellness (24). Funk (25) contends that hardiness has no appreciable regulatory impact on stress or health but rather that it directly predicts psychiatric symptoms as measured by the Beck Depression Score Questionnaire. Research on Ukrainians throughout the conflict revealed a strong correlation between mental health and toughness of personality (26). Johnsen (27) examined the association between hardiness, anxiety, and depression, as well as the indirect impacts of cohesiveness and self-efficacy during international naval operations. Thus, there are three ways to examine the connection between health and hardiness: direct, indirect, and mediation. These three effects have been supported to differing degrees by further empirical research (28, 29). As such, there is no agreement on this matter (30, 31).

*H1*: The hardiness personality positively predicts the mental health level of financially-struggling medical students.

### 1.2 Coping styles and mental health

Coping style refers to individuals’ cognitive and behavioral patterns in the face of frustration and stress (32). These patterns are mainly categorized as positive coping and negative coping. The individual’s physical and mental well-being is impacted by their adopting coping styles (33). Kessler et al. discovered that in stressful events, a lack of effective response can easily lead to depression (34). Coping style is considered a critical factor in the degree of stress response. A previous study found that positive adolescent coping was linked to better mental health, while negative coping was associated with poorer mental health (35). Besharat’s research shows that during stressful events, students who utilize active coping methods demonstrate better adaptability, improved interpersonal relationship processing, increased support from the outside world, and are more likely to experience emotions conducive to mental health (36). The Symptoms study concluded that medical students’ mental health was significantly moderated by negative coping styles, increasing the risk of mental health problems (37). At the same time, some scholars have found that engaging in harmful coping mechanisms can provide temporary relief from psychological stress and help individuals cope with adverse environments (32).

*H2*: Coping methods significantly predict the mental health level of financially-struggling medical students.

### 1.3 Coping styles as a potential mediator

There are two schools of thought about coping style studies. According to the conventional trait theory, coping mechanisms are mostly constant across various contexts and eras (38). The second viewpoint is the situation theory hypothesis, which emphasizes how the environment shapes an individual’s knowledge and is predicated on the notion that people employ various strategies in different situations (39). Folkman provided a dynamic mechanical model of human-environment interaction, known as the evaluation theory of pressure and cognition, based on the two points above of view. According to the hypothesis, personality traits and external circumstances impact coping mechanisms (40). Cross-sectional and longitudinal research supports the idea that personality factors might predict coping methods (41). For instance, adaptive coping was shown to be more efficient than maladaptive coping in research by McCare and Costa on the efficiency of coping styles in problem-solving and mood alleviation, and distinct coping styles were linked to different personality traits [5642]. Kobasa’s study has also demonstrated a connection between inventive cognitive styles, extroverted personality characteristics, and active coping techniques (21). Chen X discovered notable variations in the coping strategies of college students with different hardiness levels. In addition to having a direct correlation with mental health, hardiness also has an indirect correlation with mental health due to developed coping mechanisms (42). Chinese scholar Li SY conducted a study on the hardiness personality of university students and found that the hardiness personality had a direct predictive effect on the psychological symptoms of college students (43).

Previous studies have indicated that an individual’s capacity to adjust to psychological challenges can be improved by possessing a resilient personality and an efficient coping style (44). To a certain extent, coping strategies both influence and form an individual’s hardiness personality, and the hardiness personality itself may influence coping strategies. The capacity to handle stress is a sign of coping, and developing a support system and reaching out to others may enhance mental well-being and fortify a resilient character. Harmful emotion indulgence and escape can result in a long-term build-up of issues, resulting in severe mental health issues, which can lower tolerance to hardship and hinder the development of a resilient personality. Financial and academic pressure is more common for financially-struggling medical students than for ordinary college students, which requires psychologically solid qualities to deal with the difficulties and setbacks they face. Existing research suggests that coping styles can influence the relationship between social support and mental health through mediating effects. For example, Zou WX (45) proposed that the hardiness personality trait would significantly impact the positive response of college students in ethnically diverse areas. Although we speculate that coping styles can influence the relationship between hardiness personality and mental health, the exact role it plays in these dynamics remains to be tested.

*H3*: Coping styles mediate the relationship between the hardiness personality and the mental health level of financially-struggling medical students.

### 1.4 Gender as a potential moderator

Gender differences play an important role in both psychological and social aspects, and these factors can affect individual mental health and coping styles (46). First, gender differences are evident in terms of mental health. For example, the incidence of anxiety indicates that females are more anxious and emotionally fragile than males, while males may be more susceptible to smartphone addiction (47). Relatively, men may be more susceptible to mobile phone addiction issues (48). Secondly, gender correspondence significantly affects the choice and the mode of communication. Coping style is one of the susceptibility qualities of individuals during stress, and some studies show specific differences between males and females in choosing coping styles (49). Studies have shown that males and females adopt different strategies to cope with stress. Generally speaking, girls are more willing to talk and seek help, while men are the role symbol of responsibility and independence, and they are more willing to solve psychological problems by themselves (50). Furthermore, differences in emotional expression and processing also influence their coping strategies, and females are generally more willing to express and share their emotions, while men may choose to suppress emotions or distract attention through activity.

*H4*: Gender plays a moderating role between coping styles and mental health levels.

### 1.5 The present study

**Figure 1:**
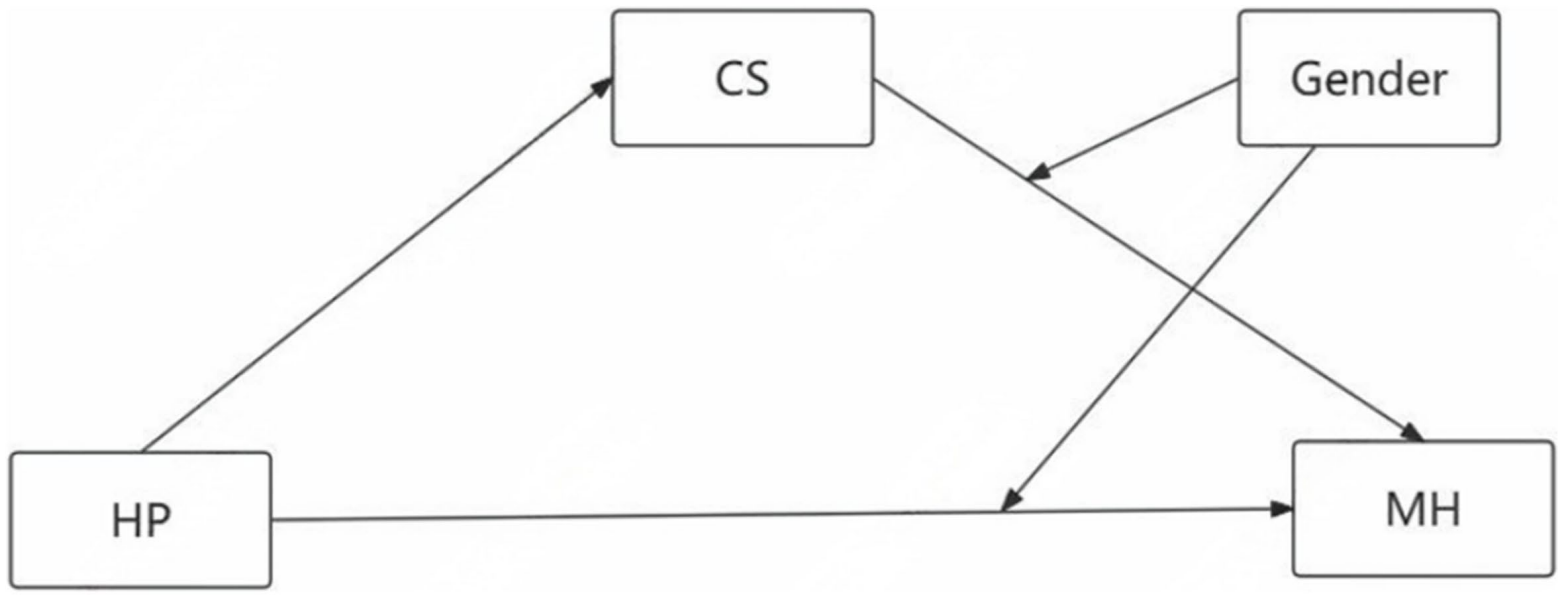
Theoretical path model. HP, hardiness personality; CS, coping style; MH, mental health.

Based on a review of the literature, we aimed to test a model that examines the relationship between hardiness personality and mental health of financially-struggling medical students, as well as its internal mechanisms, i.e., coping style and gender (Figure 1). This may help develop intervention and prevention strategies in financially-struggling medical students with psychological problems.

## 2 Materials and methods

### 2.1 Participants and procedures

The subjects of this study are medical students with a family poverty background from two private medical colleges of School of Clinical Medicine, Anhui Medical University and Anhui Sanlian College. Medical students with a family poverty background are officially recognized, including the students’ family income, consumption situation and the family poverty certificate of the local government. Therefore, it is easy to obtain the list of financially-struggling medical students in two universities, including the student name, student number, specific major name, email and other information, to ensure that each student can be identified. Second, before the test recruitment familiar with the research program of 10 staff, and guide students to fill in the questionnaire and record, before the test, the researchers and the two universities of the teacher communication, notify the two college financially-struggling medical students in the specified time arrived at the designated classroom, the staff guide students to complete the questionnaire, if there are other reasons failed to fill to the scene of the family economic difficulties students, take the form of questionnaire star online survey to complete the questionnaire, the study test a total of 1,500 people. Fourth, after the questionnaire was filled in, the staff conducted questionnaire screening and eliminated invalid questionnaires, among which 1,360 valid questionnaires were collected, with an effective rate of 90.6%. In addition, 640 valid questionnaires were randomly selected from each university by the fishbowl method, and 1,280 questionnaires were selected from the two universities for follow-up study (51). Fifth, in the process of the survey, the confidentiality and privacy of the research objects are noted, and the questionnaire is collected in an anonymous form. The collection time of the questionnaire is from December 14,2023 to February 14,2024, and the selected samples meet the requirements of the survey.

## 3 Measures

### 3.1 Subjects

The questionnaire consists of two parts. The first part collects demographic information, gender (all the genders in this study refer to the Cisgender), grade, growing environment, etc. The second part introduces the hardiness personality questionnaire, coping styles questionnaire and mental health questionnaire for financially-struggling medical students.

### 3.2 Research tools

#### 3.2.1 The hardiness personality assessment scale of college students (HPASCS)

Based on the relevant research of foreign scholars, Lu Guohua, a Chinese scholar, compiled the Hardiness Personality Scale of College Students according to the actual situation in China (52). The scale consists of 27 questions in four dimensions: hardiness, investment control and challenge. The test–retest reliability of the total scale and each scale dimension is between 0.89 and 0.92, indicating that the scale has high test–retest reliability. The scoring was performed by Likert-like 4, with four grades from 1 for “Completely out of line with” to 4 for “perfectly.” The Cronbach’s *α* of this scale was 0.908.

#### 3.2.2 Symptom checklist 90 (SCL-90)

SCL-90 is one of the world’s most famous mental health test scales. It contains 90 items that reflect on a subject’s somatization, obsessive-compulsive symptoms, interpersonal sensitivity, depression, anxiety, hostility, phobic anxiety, paranoia, psychosis, and other dimensions of their well-being, such as sleep and diet. The scoring was performed by Likert type 5, with five grades from 1 “not at all” to 5 “extremely.” Among them, the total score of 90 and the total score of less than 160 points were included in the mental health status. According to the SCL-90 scoring criteria, the higher the SCL-90 score, the lower the level of mental health. The significance of Cronbach’s *α* of the scale was 0.979, KMO was 0.970, and Bartlett sphericity test was 0.001.

#### 3.2.3 Simplified coping style questionnaire (SCSQ)

The SCSQ was compiled by Chinese scholar Xie Yanin to evaluate the subjects’ coping styles. It consists of 20 items, including two dimensions: positive coping (1–12) and negative coping (13–20) (53). The scale was graded from 0 to 3, 0 = “not,” 1 = “occasionally,” 2 = “sometimes,” 3 = “after,” and in which the subject selected the corresponding option according to their situation. The test–retest reliability of this scale is 0.89, the internal consistency coefficient *α* is 0.90, the dimension of positive response and negative response Cronbach’s α is 0.89 and 0.78, respectively, and the correlation validity of structure validity and effect standard is ideal.

### 3.3 Data analysis

SPSS 27.0 and PROCESS 3.2 macro (IBM Crop) are used to assess complex data entry and analysis models, including mediators and moderators. First, the data screening revealed no outliers or missing values. Second, descriptive statistics, correlation analysis, and independent sample *t*-tests were calculated between the primary variables. Third, we used Model 4 to investigate the mediating role of coping styles in the relationship between hardiness personality and mental health. Fourth, Model 15 examines the moderating role of gender in the relationship between coping styles and mental health. The appropriate mediation (Model 4) and gender regulatory mediation (Model 15) were tested using the 3.2 macro process developed by Hayes (54).

Model 4 usually refers to the single mediation model in Hayes ‘PROCESS macro, which means testing the influence of the independent variable (HP) on the dependent variable (MH) through the mediating variable (CS). Model 15 is a moderated mediating effect model, including an independent variable (HP), a mediating variable (CS), a regulatory variable (Gender), and a dependent variable (MH). The moderating variable (Gender) moderates the effect of the mediating variable (CS) on the dependent variable (MH), and also regulates the effect of the independent variable (HP) on the dependent variable (MH). The reason why we do not use model 6 and model 92 in the research process is that after consulting a large number of literatures, we have not found that the academic community has divided the coping style into positive coping and negative coping as a chain mediating effect (55). However, it is common for the academic community to use positive coping and negative coping as two dimensions of research (53, 56, 57). Based on 5,000 bootstrap samples, the bootstrap confidence intervals (CIs) determined whether the effects in Model 4 and Model 15 were statistically significant. The 95% bias-corrected CIs did not contain zero, indicating a significant effect. All study variables were standardized in model 4 and Model 15 before data analyses. *p*-values <0.05 were considered statistically significant.

## 4 Results

### 4.1 Basic information for each variable and testing for gender, growing environment, and grade differences

Table 1 details the means and standard deviations of each variable. A *t*-test for gender and growth environment differences for each variable showed gender differences in hardiness personality, mental health, and coping style. In contrast, the growth environment only differs in hardiness personality and not in other variables. Multivariance analysis of OVA showed differences in hardiness personality and coping styles, and no differences were found in mental health. Financially-struggling medical students from the Clinical Medical College of Anhui Medical University and Anhui Sanlian University showed differences in active coping methods (*p* < 0.01) and mental health (*p* < 0.01), respectively, with no differences in other core variables.

**Table 1:** Comparison of mean, standard deviation, and differences for gender, growth environment, and grade for each variable.

Variables		HP			PCS			NCS			MH		
		M ± SD	*t*	*p*	M ± SD	*t*	*p*	M ± SD	*t*	*p*	M ± SD	*t*	*p*
School	CMS	2.54 ± 0.56	−0.659	0.510	1.98 ± 0.49	−2.188	0.029**	1.122 ± 0.47	−1.471	0.142	1.60 ± 0.52	−2.416	0.016**
	ASU	2.56 ± 0.46			2.04 ± 0.51			1.16 ± 0.54			1.67 ± 0.53		
Gender	Male	2.68 ± 0.53	7.928	<0.001	2.04 ± 058	0.758	0.448	1.52 ± 0.68	3.667	<0.00 1	1.62 ± 0.52	−1.231	0.219
	Female	2.46 ± 0.47			2.01 ± 0.52			1.38 ± 0.65			1.65 ± 0.53		
Growth environment	Urban	2.63 ± 0.48	3.461	<0.001	2.03 ± 0.57	0.376	0.707	1.52 ± 0.72	2.538	0.011	1.67 ± 0.54	1.527	0.127
	Rural	2.52 ± 0.52			2.02 ± 0.54			1.41 ± 0.64			1.62 ± 0.52		
			F	*p*		F	*p*		F	*p*		F	*p*
Grade	Freshm an	2.55 ± 0.42	11.55 1	<0.001	1.85 ± 0.40	21.77 4	<0.00 1	1. 18 ± 0.55	38.25 0	< 0.01	1.62 ± 0.47	2.396	0.067
	Sopho more	2.42 ± 0.45			2.07 ± 0.50			1.54 ± 0.74			1.59 ± 0.45		
	Junior	2.61 ± 0.57			2.00 ± 0.56			1.36 ± 0.60			1.70 ± 0.53		
	Senior	2.63 ± 0.57			2. 18 ± 0.65			1.69 ± 0.65			1.64 ± 0.62		

HP, hardiness Personality; PCS, positive coping style; NCS, negative coping style; MH, mental health, CMS, Clinical Medical School of Anhui Medical University; ASU, Anhui Sanlian University.

*p < 0.05; **p < 0.01.

### 4.2 Correlation analysis of each variable

The results of the correlation analysis are shown in Table 2. The results showed that the hardiness personality and positive response (*r* = 0.489, *p* < 0.01), negative coping (*r* = 0.132, *p* < 0.01), and mental health and negative coping (*r* = 0.142, *p* < 0.01). There was a significant negative correlation between mental health and hardiness personality (*r* = −0.164, *p* < 0.01) and positive coping styles (*r* = −0.192, *p* < 0.01). These results supported Hypothesis 1 and Hypothesis 2.

**Table 2:** Correlations and means of each variable.

	M ± SD	HP	NCS	MH
HP	2.552 ± 0.512	1		
PCS	2.000 ± 0.493	0.489**		
NCS	1.431 ± 0.637	0. 132**	1	
MH	1.637 ± 0.525	−0. 164**	0. 142**	1

*p < 0.05; **p < 0.01.

### 4.3 Mediating effects test

In the section on testing for the mediation effect, this study utilized the mediation variable test method proposed by Baron and Kenny to examine whether coping styles mediate between hardiness personality and mental health (58). The study followed three steps: first, to determine whether hardiness personality significantly affects coping styles; second, to investigate whether hardiness personality significantly affects mental health; and third, to assess whether coping styles significantly impact mental health. If the first three steps yield positive results, the study will proceed to test whether coping significantly impacts mental health. If the influence of a hardy personality on mental health weakens or becomes insignificant, then the coping style is confirmed as playing an intermediary role. The specific empirical study results can be found in Table 3.

**Table 3:** Analysis of the mediating effect between hardiness personality, mental health and coping style.

Variables		Overall fit index			Significance of regression coefficients	
Outcome variables	Predictors	*R* ^2^	^*R*^2^	F	Beta	*t*
MH	HP	0.027	0.026	35.452**	−0.169**	28.045**
PCS	HP	0.239	0.238	401.528**	0.471**	20.038**
NCS	HP	0.017	0.017	22.532**	0.164**	4.747**
MH	HP	0.043	0.042	28.969**	−0.095**	−2.948**
	PCS				−0.156**	−4.680**
	NCS				0.138**	6.083**

*p < 0.05; **p < 0.01.

According to the analysis in Table 3, hardiness personality negatively predicted the mental health of family poverty medical students (*β* = −0.169, *p* < 0.01). The hardiness personality is still a significant negative predictor of mental health (*β* = −0.095, *p* < 0.01). Hardiness personality had a significant positive prediction of positive coping (*β* = 0.471, *p* < 0.01) and negative coping (*β* = 0.164, *p* < 0.01). Positive coping style of financially-struggling medical students had a significant negative prediction of mental health level (*β* = −0.156, *p* < 0.01), while negative coping style had a significant positive prediction of mental health level (*β* = 0.138, *p* < 0.01).

The coping methods and the regulatory effects of each dimension are shown in Table 4. The results showed that positive and negative coping styles significantly mediated between hardiness personality and mental health, respectively, with both pathways accounting for 52.594% of the total effect with a total indirect effect of 0.056. The mediation effect consists of two pathways of indirect effects: the indirect effect pathway 1 (hardiness personality → positive coping style → mental health) accounted for 43.705% of the total effect, and the effect value was 0.033; Indirect effect path 2 (hardiness personality →negative coping style →mental health) accounted for 11.799% of the total effect, and the effect value was 0.023. The effect values of the two indirect pathways were significant (95% confidence intervals excluding 0). This also indicates that the impact of a hardy personality on the mental health of financially-struggling medical students will be related to different coping styles. These results supported Hypothesis 3.

**Table 4:** Analysis of coping style and the mediating effects of the dimensions.

Categories	Effect value	Boot estimate	LLCI	ULCI	Percentage
Total effect	−0.169	0.028	−0.224	−0.113	
Direct effect	−0.080	0.029	−0.137	−0.023	47.337%
Mediating effect 1	−0.074	0.033	−0.103	−0.039	43.705%
Mediating effect 2	0.023	0.023	0.011	0.034	11.799%
Total indirect effects	−0.089	0.056	−0.111	−0.064	52.594%

### 4.4 Analysis of the moderating effect of gender

**Figure 2:**
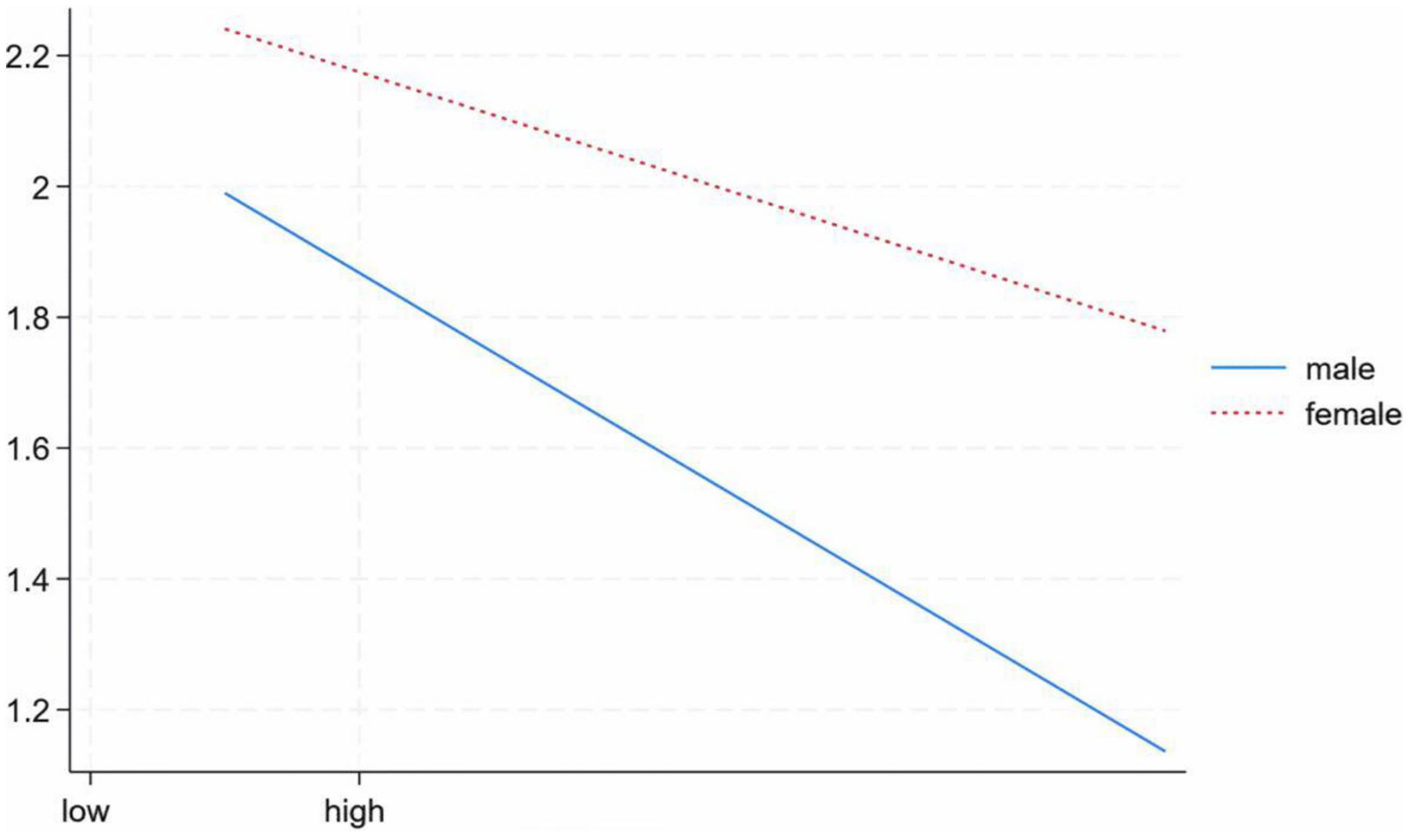
Moderating effect of gender on the relationship between positive coping style and mental health.

The moderating effects of sex are shown in Table 5. The interaction term of the positive coping style (*β* = 0.115, SE = 0.053, 95% CI: 0.012 to 0.218) and gender significantly impacted mental health, indicating that gender played a moderating role in the relationship between positive coping and mental health. A simple slope test (Figure 2) found that with the enhancement of active coping, mental health symptoms were declining, and the level of mental health was improving. With the enhancement of active coping, the effect of gender on mental health symptoms among financially-struggling medical students was significantly higher in males (*β*simple slope = −0.244, *p* < 0.01) than in females (*β*simple slope = −0.132, *p* < 0.01). This indicates that the more positive the coping style, the mental health level of men among poor medical students will be higher than that of women. These results supported Hypothesis 4.

**Table 5:** The moderating effects of gender.

	MH				*R* ^2^	F
	*β*	SE	*t*	*p*		
PCS	−0.318	0.085	−3.718	0.000	0.047	15.885
Gender	−0.221	0.111	−1.990	0.047		
PCS*Gender	0.115	0.053	2.181	0.029		
NCS	−0.022	0.071	−0.308	0.758	0.056	18.998
Gender	−0.128	0.070	−1.837	0.066		
NCS*Gender	0.096	0.043	2.202	0.028		

## 5 Discussion

### 5.1 Relationship between hardiness personality and mental health

This study found that the hardiness personality has a negative impact on mental health symptoms and a positive effect on a psychological level, consistent with the findings of Chinese scholar Li Guomin on the status of the hardiness personality of college students (59). Norwegian scholar Reknes (60) believes that high resilience plays a buffer role in the relationship between bullying and anxiety, because individuals with high resilience do not experience higher levels of anxiety when facing bullying. This suggests that the positive effects of hardiness personality on mental health levels is cross-cultural. Individuals with high hardiness are characterized by intense feelings of control, challenge, and commitment (61). Individuals with high hardiness tend to believe in their ability to control or influence the entire process. They view challenging new events as opportunities for personal growth and learning, have positive beliefs, and can quickly recover and grow in adversity (62). This study found that the higher the hardiness level, the higher the mental health level among financially-struggling medical students. This may be because a hardy personality can improve achievement motivation among financially-struggling medical students (63). In addition, under the influence of Chinese traditional culture, individuals with highly hardy personality tend to succeed and failure to internal factors, efforts and mentality, and the major success and failure due to the environment, luck, tasks and other external factors, this attribution may enhance individual control of the future, to cultivate a positive attitude to cope with the challenges of the outside world.

### 5.2 The mediating role of coping methods

This study found that coping styles play a mediating role in the relationship between hardiness personality and mental health level among financially-struggling medical students. Among them, the impact of positive coping is more significant than that of negative coping. Relevant studies have proved that individuals with hardiness personality are tenacious and can deal with the challenges in life (64). How should we encourage financially-struggling medical students to establish a hardy personality? Coping styles can play an essential role in this regard. Positive coping style as a stabilizing factor can help individuals maintain positive psychological qualities during stressful periods (65). Meanwhile, positive coping was associated with positive outcomes, and individuals with positive coping styles tended to be more likely to achieve positive outcomes such as higher life satisfaction, better mental health status and higher subjective well-being (66, 67, 75). In contrast, negative coping styles tend to be associated with negative outcomes, such as higher levels of anxiety and depression (68). Students in private colleges respond to challenges through a positive attitude and tenacious character, and due to the orientation of national policies, they do not have more school resources to solve problems and usually need to pay higher tuition fees and obtain better educational resources and employment opportunities. At the same time, financially struggling medical students in private colleges and universities experience family-related stressors, such as housing pressure, sudden accidents, parental divorce, family conflict, and other adverse situations, which often have problems such as self-blame, escape, and fantasy (69). Active response methods can change the situation through positive cognition and action efforts, as well as calm, rational, and thoughtful solutions (70).

### 5.3 The moderating role of the gender

This study found that male hardiness personality, coping style and mental health were significantly higher than girls. The latter half of the mediating effects of hardiness personality affecting mental health levels through positive coping styles is modulated by gender. Hardiness personality is not significant to moderate the second half of mental health by gender. According to the theory of gender role socialization, since males and females play different roles and assume different tasks in the socialization process, they will have different performances in coping methods (71). Carter reported that male students are more mature than female students in terms of coping methods (72). When facing problems, women prefer more internal strategies, and men prefer more external strategies (73). At the same time, with the increase in positive coping styles, men will respond more actively than women. For example, Chinese scholar Tao BL believed that with increased physical exercise, men will respond more actively than women and can improve mental health problems (74).

## 6 Conclusion

This study focuses on the relationship between hardiness personality and mental health, reveals the important role of coping in this process, for global researchers and educators to further explore how to improve the mental health problems of higher education students, emphasizing the key role of individual traits in mental health maintenance. The global introduction of the association between hardiness personality and mental health into the Chinese context helps enrich the theoretical framework in international psychology.

The mental health problems of financially-struggling medical students require the joint efforts of universities and families. Medical schools should focus on creating a competitive environment and cultivating financially-struggling medical students to form a hard personality and develop good mental health. Families need to play the function of emotional support, giving children warmth, understanding and encouragement, helping them establish a positive emotional state and enhancing psychological resilience. In addition, both families and schools should pay attention to reducing gender differences, pay more attention to women in financially-struggling medical students, and jointly promote the good mental health status of financially-struggling medical students. This study may provide a reference for researchers and educators worldwide to explore further how to improve students’ mental health problems in higher education. Cultivating a high-hardiness personality is conducive to financially-struggling medical students actively dealing with negative life events to form a healthy psychological state. However, this study has limitations.

Although this study extends the results of previous studies, some shortcomings remain. First, the study population was based on financially-struggling medical students from medical schools in eastern China, generalizing the results of this study limited. In the future, the sample selection in the eastern and western regions of China can be increased to improve the representativeness of the sample research results. Second, despite our steps to mitigate medical students’ concerns by highlighting the confidentiality of the results, the study may have response bias. Future studies should incorporate experimental methods for measuring the variables. Third, this study focused on the relationship between overall hardiness personality and mental health, but did not specifically investigate the relationship between gritty personality and mental health dimensions and whether differences exist between each dimension, adding future research. Fourth, methodologically, a cross-sectional design limits our capture of variation between variables, and longitudinal or dynamic studies could be considered in the future.

## Data Availability

The original contributions presented in the study are included in the article/Supplementary material, further inquiries can be directed to the corresponding author.
